# The Response of Heterotrophic Prokaryote and Viral Communities to Labile Organic Carbon Inputs Is Controlled by the Predator Food Chain Structure

**DOI:** 10.3390/v9090238

**Published:** 2017-08-23

**Authors:** Ruth-Anne Sandaa, Bernadette Pree, Aud Larsen, Selina Våge, Birte Töpper, Joachim P. Töpper, Runar Thyrhaug, Tron Frede Thingstad

**Affiliations:** 1 Department of Biology, University of Bergen, N-5020 Bergen, Norway; Bernadette.Pree@uib.no (B.P.); selina.vage@bio.uib.no (S.V.); Birte.topper@bio.uib.no (B.T.); frede.thingstad@bio.uib.no (T.F.T.); 2 Uni Research Environment, Nygårdsgaten 112, 5008 Bergen, Norway; aud.larsen@bio.uib.no; 3 Norwegian Institute for Nature Research, Thormøhlensgate 55, N-5008 Bergen, Norway; joachim.topper@nina.no

**Keywords:** marine viral diversity, viral–host interaction, high latitude microbes, minimum food web model, copepods, ciliates, nutrient limitation, trophic cascade

## Abstract

Factors controlling the community composition of marine heterotrophic prokaryotes include organic-C, mineral nutrients, predation, and viral lysis. Two mesocosm experiments, performed at an Arctic location and bottom-up manipulated with organic-C, had very different results in community composition for both prokaryotes and viruses. Previously, we showed how a simple mathematical model could reproduce food web level dynamics observed in these mesocosms, demonstrating strong top-down control through the predator chain from copepods via ciliates and heterotrophic nanoflagellates. Here, we use a steady-state analysis to connect ciliate biomass to bacterial carbon demand. This gives a coupling of top-down and bottom-up factors whereby low initial densities of ciliates are associated with mineral nutrient-limited heterotrophic prokaryotes that do not respond to external supply of labile organic-C. In contrast, high initial densities of ciliates give carbon-limited growth and high responsiveness to organic-C. The differences observed in ciliate abundance, and in prokaryote abundance and community composition in the two experiments were in accordance with these predictions. Responsiveness in the viral community followed a pattern similar to that of prokaryotes. Our study provides a unique link between the structure of the predator chain in the microbial food web and viral abundance and diversity.

## 1. Introduction

Microorganisms are the main controllers of biomass and energy fluxes in the ocean. Together with their viruses, they form a tightly linked web of trophic interactions at the base of marine food webs, typically connected to the higher part of the food chain with multicellular organisms through copepod predation on microbes. Within the microbial part of this ecosystem, the composition and activity of the community of pelagic heterotrophic prokaryotes are presumably shaped both by bottom-up factors such as the availability of mineral and organic-C nutrients, and by the top-down mechanisms of predation [1] and viral lysis [2,3,4]. Nutrient availability and predator control have to a large extent been studied using a “black box” approach, treating the heterotrophic prokaryotes as one plankton functional type (PFT), disregarding internal community composition and differences in activity between community members [5,6], and leaving us with a limited understanding of how population dynamics at the two levels of resolution are connected. Top-down control by viruses is believed to be more specific than predatory control, regulating the size of specific host groups, and thus acts more on community composition than on community size [7]. With host-specific viruses, host–virus interactions must, however, work both ways so that a change in host community composition will be reflected in a subsequent change in viral composition. 

In the present study, we approach these interactions by revisiting the study by Larsen et al. [8], who demonstrated the explanatory power of a black-box modeling approach in their analysis of contrasting food web level responses observed in two similarly bottom-up perturbed mesocosm experiments (Polar Aquatic Microbial Ecology) (PAME)-I and PAME-II). Larsen et al. [8] were able to explain these contrasting responses using a “minimum” food web model, consisting of six PFTs (Figure S1). An essential conclusion of their study was that food web level responses to the bottom-up manipulations applied (glucose and mineral nutrients) were strongly modulated by the different states of the trophic cascade, from copepods via ciliates and heterotrophic nanoflagellates (HNF) to heterotrophic prokaryotes, essentially identifying the seasonal vertical migration of Arctic copepods as an ultimate cause in the cause–effect chain of such a trophic cascade. Larsen et al. [8] did not include any resolution of the prokaryote community, or any representation of viruses, in their analysis. 

**Figure 1:**
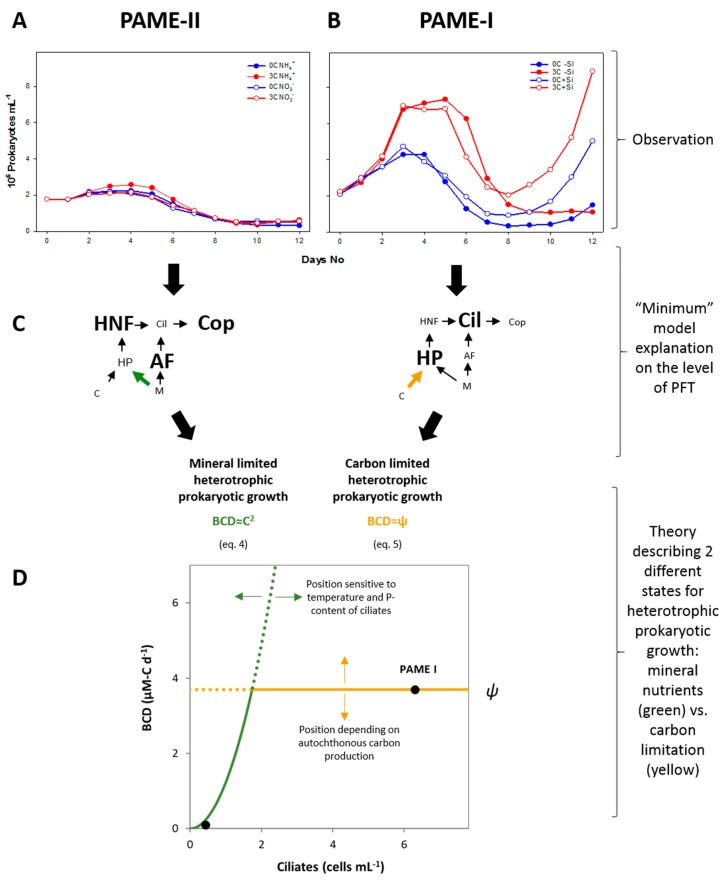
Different heterotrophic prokaryote (HP) responses to glucose additions ((**A**,**B**) data from [8],) are predicted qualitatively by the model (**C**) that links the type of growth rate limitation of bacteria to ciliate abundance (**D**) [8]. (**A**) Observed response in PAME-II was negligible, while (**B**) observed response in PAME I was a strong effect of glucose addition. (**C**) The pentagon food web structure (see also Figure S1, left) has two possible limiting states for heterotrophic prokaryotic growth: (**D**) either free mineral nutrients limitation (MNL), where bacterial carbon demand increases as the square of ciliate biomass increases (green curve); or organic carbon limitation (OCL), where bacterial carbon demand is equal to the supply (*ψ*) of degradable organic-C (yellow line). Since bacterial carbon demand (BCD) cannot sustainably exceed the supply, the shift between the two states occurs at the ciliate density where the green and yellow curves cross. HNF: heterotrophic nanoflagellates; AF: autothrophic flagellates; Cop: copepodes.

We here address the observation that the heterotrophic prokaryote (Figure 1A,B) and virus communities responded to labile C-addition in PAME-I, but lacked such a response in PAME-II. We hypothesize that these differences within the prokaryote and virus communities arose from the different states of the trophic cascade, as inferred by Larsen et al. [8]. Using a simplified steady state analysis of the “minimum” model, we explain how low versus high initial ciliate abundances drive model heterotrophic prokaryote growth towards mineral nutrient (MN) versus organic carbon (OC) limitation, respectively (Figure 1C). A C-limited community is presumably more responsive to the addition of glucose than an MN-limited community, replete in organic-C substrates, and the model predictions are thus in qualitative accordance with the observed differences in prokaryote community composition response. This interpretation extends the cascading effect of seasonally migrating Arctic copepods from the microbial food web level, as suggested by Larsen et al. [8], to the level of internal community structures of the prokaryote, and even the viral communities. 

## 2. Materials and Methods

### 2.1. Model Framework

We used the minimum microbial food web model (Figure S1) to explain observed responses and derive a theory for different states of limitation (Figure 1). The dynamic version of the minimum model accurately predicts mesocosm responses in different environments [5,8]. Here we use a simplified steady-state analysis of the model. The trophic interactions between ciliates, heterotrophic nanoflagellates, prokaryotes, and autotrophic flagellates (Figure 1C,D and Figure S1, left) reveal a link between ciliate abundance and bacterial carbon demand (Equations (4)–(6)), as described in the following. Under steady state, mass balance must be fulfilled such that “growth = loss” for each state variable. Assuming that food consumption is proportional to food concentration, heterotrophic flagellates balance their growth by their loss as follows (explanation of symbols is found in Table 1):

(1) $$Y_{H}\alpha_{H}BH=\alpha_{C}HC.\text{ Solving this for B gives }B=\frac{\alpha_{C}}{Y_{H}\alpha_{H}}C.$$

For autotrophic flagellate, growth = loss is given by:

(2) $$\alpha_{A}PA=\alpha_{C}AC.\text{ Solving this for P gives }P=\frac{\alpha_{C}}{\alpha_{A}}C.$$

For phosphate-limited growth of heterotrophic prokaryotes, growth is expressed as $\mu =\alpha_{B}P$, which, by insertion from Equation (2), gives:

(3) $$\mu =\frac{\alpha_{B}\alpha_{C}}{\alpha_{A}}C.$$

Steady-state prokaryote carbon demand under P-limitation (*BCD_P_*) is given by the ratio of production (μB) over yield $\left(Y_{BC}\right)$. Assuming yield ($Y_{BC}$) to be a constant and independent of the growth rate *μ*, *BCD_P_* can from inserting Equations (1) and (3) be expressed in terms of ciliate biomass as:

(4) $$BCD_{P}=\frac{\mu B}{Y_{BC}}=\frac{\alpha_{B}\cdot \alpha_{C}^{2}}{Y_{BC}Y_{H}\alpha_{H}\alpha_{A}}C^{2}.$$

This gives a quadratic relationship between prokaryote carbon demand and ciliate abundance (Figure 1, MNL state). Prokaryote carbon demand can, however, not exceed the rate *ψ*, at which labile DOC is supplied by autochthonous and/or allochthonous sources*.* For high ciliate abundances giving $BCD_{P}>\psi$, the system thus shifts to C-limited prokaryote growth with prokaryote carbon demand (OCL state in Figure 1):

(5) $$BCD_{C}=\Psi .$$

Equations (4) and (5) can be summarized as:

(6) $$BCD=\min\left(\frac{\alpha_{B}\cdot \alpha_{C}^{2}}{Y_{BC}Y_{H}\alpha_{H}\alpha_{A}}C^{2},\Psi \right),$$

where the left entry in the bracket describes BDC under mineral nutrient limitation, and the right entry describes BDC under carbon limitation (Figure 1). Using a conversion factor of P-content per ciliate (*σ*) and optimizing the model for the PAME experiments by assuming a temperature sensitivity of the rates with a Q_10_ ≈ 1.3 [8], this adjusts Equation (6) (shown in Figure 1B) to:

(7) $$BCD=\min\left(\frac{\alpha_{B}\cdot \alpha_{C}^{2}\sigma^{2}}{Y_{BC}Y_{H}\alpha_{H}\alpha_{A}Q_{10}}C^{2},\Psi \right),$$

where ciliate abundance *C* is in cells mL^−1^ and the parameters are listed in Table 1.

To obtain the estimate of *σ*, we assumed a specific carbon content of 0.13 pg-C μm^−3^, Redfield stoichiometry (molar C:P = 106:1), and an equivalent spherical diameter of 20 μm [9].

### 2.2. Experimental Design

#### 2.2.1. Mesocosm Setup

**Figure 2:**
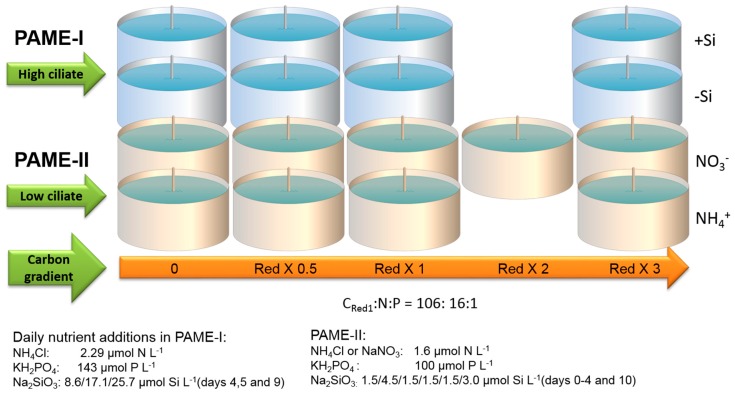
Experimental design of the two mesocosms, (Polar Aquatic Microbial Ecology) PAME-I (2007) and PAME-II (2008). Both experiments received the same dose of nitrogen (N) and phosphorus (P) in Redfield ratio (C:N:P = 106:16:1 molar). Glucose was added in two four-point carbon addition-gradients (0, 0.5, 1, 3 × Redfield in glucose) in PAME–I and one of the series in PAME-II. The other series in PAME-II was a five-point carbon addition-gradient (0, 0.5, 1, 2, 3 × Redfield in glucose C). Samples for this study were taken from the treatments with 0 and 3 × Redfield carbon additions. In PAME-I, nitrogen was added as NH_4_Cl. In PAME-II, nitrogen was added as NaNO_3_ in the NO_3_^−^ gradient and as NH_4_Cl in the NH_4_^+^ gradient. In PAME-I, silicate was added to the +Si units only. In PAME–II all tanks were kept silicate-replete. More information about the experimental setup can be found in [8].

In PAME-I eight units (each 700 L) formed two four-point gradients of additional DOC in the form of glucose (0, 0.5, 1 and 3 times Redfield ratio in terms of carbon relative to the nitrogen and phosphorus additions) (Figure 2). All eight units also got a daily dose of NH_4_^+^ and PO_4_^3−^ in Redfield ratio. Two gradients were set up, one with silicate addition [8]. Samples for flow cytometric counts of viruses were collected from all treatments every day. Samples for DGGE and PFGE were collected from four of the tanks: 0 and 3C without Si (−Si) treatment; 0 and 3C with Si treatment (+Si). 

In PAME-II all nine units (each 900 L) formed two four-point gradients of additional DOC in the form of glucose (0, 0.5, 1, 2 and 3 times Redfield ratio in terms of carbon relative to nitrogen and phosphorus additions) (Figure 2). The two gradients in glucose were kept silicate-replete. NH_4_^+^ was used as the dissolved inorganic nitrogen (DIN) source in one gradient (units 1 to 5) and NO_3_^−^ in the other (units 6–9). All units got a daily dose of PO_4_^3−^ in Redfield ratio. Flow cytometric counts of viruses and bacteria were performed on samples from all treatments, whereas DGGE and PFGE was performed on samples from four of the tanks: 0; 3C with NH_4_^+^ treatment; and 0, 3C with NO_3_^−^ treatment. For a more detailed description of the PAME-I and PAME-II setup, see [8,10].

#### 2.2.2. Viral Counts

Total number of viruses was determined with a FACSCalibur flow cytometer (Becton–Dickinson, FACSCalibur, Biosciences, Franklin Lakes, NJ, USA) equipped with an air-cooled laser providing 15 mW at 488 nm and with standard filter set-up. Enumeration of virus-like particles was performed on samples fixed with glutaraldehyde (final concentration 0.5% *v*/*v*) prior to staining with 1 × SYBR Green I (Molecular Probes, Eugene, OR, USA). A minimum of two different dilutions per sample were counted for 60 s each at a viral event rate between 100 and 1000 s^−1^. Blanks (Tris EDTA (TE) buffer and SYBR Green) were run at regular intervals and subtracted from the total number in order to ensure instrument noise was not being counted as viral particles. The flow cytometer instrumentation and general methodology followed the recommendations of Marie et al. [11]. 

#### 2.2.3. Viral Concentration and Pulse Field Gel Electrophoresis (PFGE)

Two liters of seawater per sample were pre-filtered through a 142 mm diameter 1.2 μm pore-size low-protein-binding Durapore membrane filter (Millipore Corp., Billerica, MA, USA) to remove larger particles/microorganisms from the sample. The virus-containing filtrates were further concentrated to a final volume of approx. 50 mL by tangential flow filtration using a QuixStand benchtop system equipped with a 100,000 pore size (NMWC) hollow fibre cartridge (GE Healthcare Bio-Sciences AB, Uppsala, Sweden) [12]. Recovery of the viruses using this approach has been measured to be 40–60% (Sandaa, personal observation). Viral particles were subsequently concentrated by ultracentrifugation (Beckman L8-M with SW-28 rotor) for 2 h at 28,000 rpm at 10 °C. The viral pellet was dissolved in 200 μL of SM buffer (0.1 M NaCl, 8 mM MgSO_4_ × 7H_2_O, 50 mM Tris-HCl, 0.005% *w*/*v* glycerine). 

PFGE was performed according to Sandaa et al. [13]. Four viral agarose plugs were prepared from the 200 μL viral concentrate for PFGE. The plugs were made immediately after viral concentration and stored in a TE buffer (20:50) at 4 °C and analyzed within a month after sampling. The samples were run on a 1% *w*/*v* SeaKem GTG agarose (FMC, Rockland, ME, USA) gel in 1 × TBE gel buffer using a Bio-Rad DR-II CHEF Cell (Bio-Rad, Richmond, CA, USA) electrophoresis unit. From each sample we used three of the plugs and ran them at three different pulse-ramp conditions in order to separate a large range of viral genome sizes [3,13]. Gels were visualized and digitized using the Fujifilm imaging system, LAS-3000. 

#### 2.2.4. DNA Isolation, PCR, and Denaturing Gradient Gel Electrophoresis (DGGE)

Depending on the biomass in the different units, 50–250 mL of water was filtered through a sterile 0.2 μm polycarbonate filter. The filters were flash-frozen in liquid nitrogen and stored for approx. two months before further analysis. DNA isolation, PCR, and DGGE were performed as described in Töpper, et al*.* [14]. The DGGE of samples from PAME-II was analyzed with a denaturing gradient of 20–60% and an internal standard consisting of 16S rDNA amplicons of four bacteria isolates: *Gelidibacter algens* (DSMZ: 12408), *Microbacterium maritypicum* (DSMZ: 12512), *Flexibacter aurantiacus* (DSMZ: 6792), and *Sulfitobacter mediterraneus* (DSMZ: 12244) [14]. 

#### 2.2.5. Statistical Analysis 

Correlation between ciliate abundance [8] and virus to prokaryote ratio (VPR) was investigated by calculating the Pearson correlation coefficient (*ρ*). Digitized images of DGGE (bacteria) and PFGE (virus) were analyzed using the programs Gel 2K (Svein Norland, Dept. of Biology, University of Bergen, Norway) and Bionumerics 4.5 (Applied Maths, Sint-Martens-Latem, Belgium), respectively, which determine the presence and absence of bands (fingerprints). Binary data of the DGGE analysis from PAME-I [15] and PAME-II (this study) together with the binary data of the PFGE analysis were compared using the program R 3.3.1 (R Development Core Team 2016). Non-metric multidimensional scaling (NMDS) was performed by using the function metaMDS in the R package vegan [16] applying the Jaccard similarity coefficient. 

Correlation between bacteria and virus ordinations of each treatment was calculated using the Procrustes statistic (R package vegan) [16]. A permutation test with 1000 repetitions of the NMDS and Procrustes statistics was performed to obtain reliable *p-*values. Permutational Multivariate Analysis of Variance (PERMANOVA, 99999 permutations) of band patterns in the DGGE and PFGE analysis were used to test whether the bacterial and viral communities grouped according to nutrient treatment (function “Adonis” R package vegan).

## 3. Results

### 3.1. Theoretical Considerations 

Combining the Lotka–Volterra equations for growth of prey communities of ciliates (i.e., AFs and HNFs, Figure 1) assuming steady-state and phosphate limitation, leads to a carbon demand for heterotrophic prokaryotes (*BCD_P_*) that scales approximately to the second power of ciliate (C) abundance (*BCD_P_* ~ *C*^2^) (see Section 2.1 for assumptions and arguments). The relationship *BCD_P_ ~ C*^2^ is illustrated by the green curve in Figure 1D. *BCD_P_* increases rapidly with ciliate abundance, but since the consumption of labile organic material cannot sustainably exceed its autochthonous production (*ψ*, horizontal line in Figure 1D), heterotrophic prokaryotes will become C-limited when *BCD_P_* > *ψ*. A closed system will therefore have a steady state with mineral nutrient-limited (MNL) bacterial growth at low ciliate abundances with a shift towards organic carbon limited (OCL) heterotrophic prokaryote growth at high ciliate abundances (Figure 1D). Supplying allochthonous labile organic-C to an OCL system is expected to evoke a marked response, compared to no response when adding it to a system in the MNL state. Although the slope of the quadratic function *BCD_P_* ~ *C^2^*, the position of *ψ*, and therefore the transition from MNL to OCL state are subject to several assumptions, they are consistent with the experimental results from Larsen et al. [8]. Interpreting the nutrient manipulated mesocosms as upscaled bioassays, the lack of response in prokaryote abundance to glucose addition indicates MNL bacterial growth in PAME-II (Figure 1A, data from [8]), in contrast to PAME-I (Figure 1B), where the strong positive response indicates OCL bacterial growth. 

### 3.2. Experimental Results

#### 3.2.1. Total Viral Abundance

**Figure 3:**
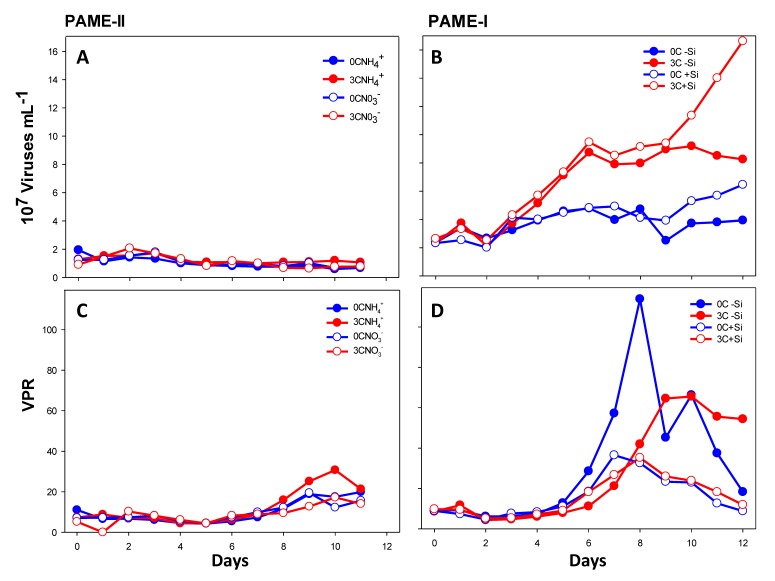
A and B, total abundance of viruses (all groups). C and D, virus to prokaryote ratio (VPR) determined by flow cytometry (FCM) of small viruses and prokaryotes. Blue circles show the data of tanks with no addition of carbon in the form of glucose, while red circles show three Redfield additions of carbon. Open and filled circles display perturbations with different nitrogen sources (open: NO_3_; filled: NH_4_) in PAME-II and with or without silicate (open: no Si addition; filled: Si addition) in PAME-I. See Figure 2 for more information about experimental design. Viral abundances during PAME-I have been previously published in Töpper et al. [15].

In general, the total viral abundance was lower in all treatments in PAME-II than in PAME-I (Figure 3A,B). Viral abundance varied between 0.9 and 2.0 × 10^7^ particles mL^−1^ in PAME-II in the start of the experiment, whereas the initial abundances in PAME-I were slightly higher (2.4 × 10^7^ mL^−1^). In PAME-II, we observed no major differences in viral abundance between the different tanks; and the abundance remained at the same level during the whole experimental period. In PAME-I, the viral abundance in tanks receiving glucose (3C−Si and 3C+Si) increased to peak values on day 6 (8.7 and 9.5 × 10^7^ particles mL^−1^, respectively). We observed a second increase in tank 3C+Si starting on day 9 and culminating on day 12 (1.7 × 10^8^ particles mL^−1^). In the two tanks without glucose addition, the viral abundance increased slightly throughout the entire experiment, reaching 6.5 and 3.9 × 10^7^ particles mL^−1^, respectively, on day 12. 

**Figure 4:**
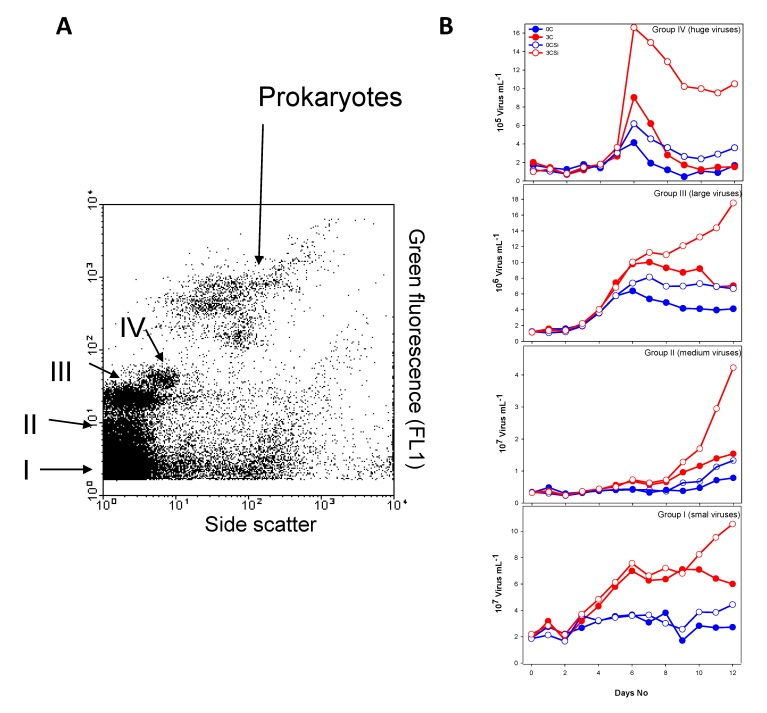
Biparametric flow cytrometry virus plots. Four different viral populations were discriminated combining side scatter signal vs. green fluorescent signal after staining with SYBR Green I (reflecting the amount of DNA and hence genome sizes): I, Small viruses, II, medium viruses, III, large viruses, IV, huge viruses (**A**) (see also Figure S2). Abundance of small (I), medium (II), large (III), and huge (IV) viruses determined by FCM in PAME-I (**B**). Blue circles show no addition of carbon in the form of glucose, while red circles show three Redfield additions of carbon. Open and filled circles display perturbations with or without silicate (open: no Si addition; filled: Si addition). See Figure 2 for more information about experimental design.

The viral particles were grouped into four populations on the basis of side scatter signal vs. green fluorescent signal after staining with SYBR Green I in PAME-I: I, II, III, and IV (I = small viruses, II: medium viruses, III: large viruses, IV: huge viruses (Figure 4A and Figure S2)). Both side- and fluorescent signals are indicators of size. Larger surface area will give a stronger scatter signal and bigger viral particles will have more DNA, which will be reflected in the more intense green fluorescence signal after staining with SYBR Green. The signals do not, however, give a basis for accurate size measurements as both granularity and surface properties affect signal strength. In PAME-II all viruses belonged to I + II (results not shown). In PAME-I, the dynamics of the various viral size groups all followed a pattern that was similar, but not identical, to that of total viruses, with low initial concentrations followed by a proliferation around day 3–5. This was most evident in the tanks receiving glucose, especially 3C+Si (Figure 4B). Group IV (huge viruses) peaked at day 6 in all tanks, with the highest concentrations in 3C+Si (1.7 × 10^6^ particles mL^−1^). 

The abundance of group III (large viruses) increased from day 3 to day 6 in all tanks and continued to increase in tank 3C+Si, reaching 1.8 × 10^7^ particles mL^−1^ on day 12. Group II (medium sized viruses) increased only slightly in numbers during the experimental period, except in tank 3C+Si, where we observed a pronounced increase starting at day 8, with 7.2 × 10^6^ particles mL^−1^ reaching 4.2 × 10^7^ particles mL^−1^ on day 12. The concentration of viruses belonging to group I (small viruses) was highest in the two glucose-amended tanks, with peaks on day 6. In 3C+Si, the concentration of this viral group increased again from day 9 to day 12, reaching a final concentration of 1.1 × 10^8^ particles mL^−1^. 

Viral to prokaryote ratio (VPR) was calculated using the abundance of viruses in group I (small viruses mainly infecting prokaryotes [17]) and total prokaryotes, determined by FCM [8]. Initial VPR was approx. 10 in both experiments, and in general VPR varied more in PAME-I (Figure 3C,D) than in PAME-II. The highest ratio (114) was detected in 0C−Si of PAME-I on day 8. The VPR showed a large and significant positive correlation with ciliate numbers (Table 2) for all treatments except PAME-I tank 0C where a non-significant and slightly negative (−*ρ* = 0.175, *p* = 0.678) association was measured. 

#### 3.2.2. Bacterial and Viral Community Structure 

**Figure 5:**
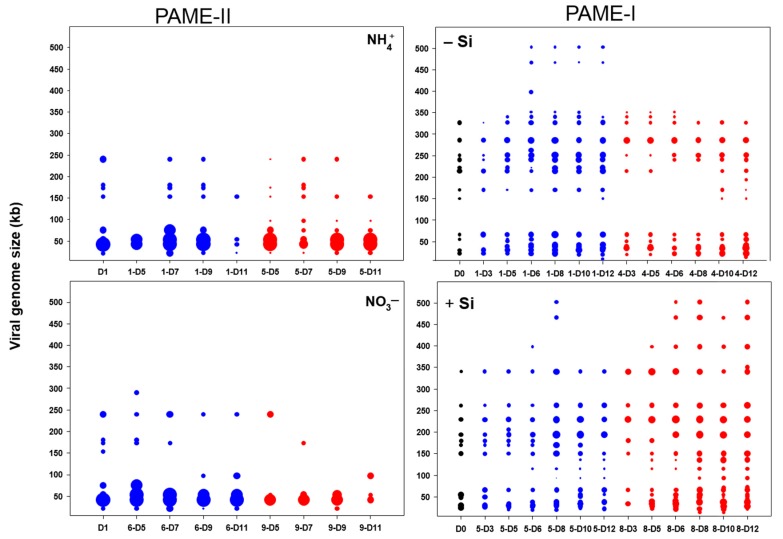
Schematic outline of the PFGE bands sorted by genome size during the mesocosm experiment in PAME-II and PAME-I. D: sampling day. The outline is based on three different electrophoresis runs for each viral sample. Blue circles show samples from treatments with no addition of carbon in the form of glucose, while red circles show samples from treatments with three Redfield additions of carbon. See Figure 2 for more information about experimental design.

**Figure 6:**
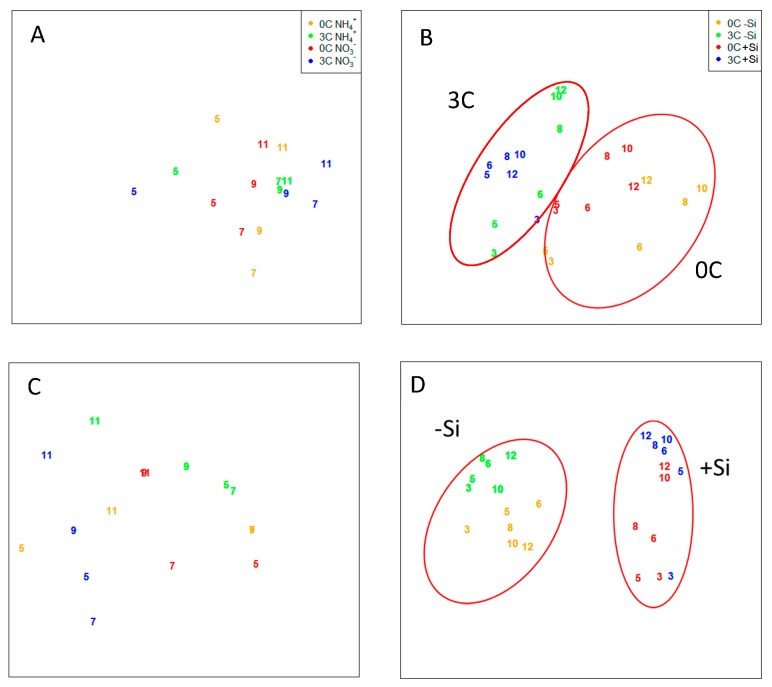
Nonmetric multidimensional scaling analysis (NMDS) based on the band pattern in the DGGE (bacteria (**A**,**B**)) and PFGE (virus (**C**,**D**)) analysis. Binary data for NMDS analysis from PAME-I is redrawn from [15]. See Figure 2 for more information about experimental design.

Thirty-one pulsed field gel electrophoresis (PFGE) bands, with genome sizes ranging from 14 to 502 kb (Figure 5), were detected in PAME-I. The number of bands was highest in the 3C+Si tank, with an increase from 11 on day 0 to 20 on day 12. Viruses with genomes larger than 350 kb were also more dominant (highest pixel value) in tank 3C+Si than in the others. During PAME-II, a total of 11 unique PFGE bands were detected ranging in size from 22 to 352 kb, with a dominance of bands between 42 and 54 kb. For PAME-II, the non-metric multidimensional scaling (NMDS) analysis based on the denaturing gradient gel electrophoresis (DGGE) and PFGE band patterns (Figure 6A,C) did not reveal any grouping. Furthermore, there was no significant support (*p* > 0.05) (Table 3) of grouping in either the DGGE nor PFGE patterns due to glucose addition, nor for co-occurring changes in bacterial host and viral communities (*p* > 0.05, Table 4. In PAME-I, both the bacterial and viral community structure changed over time and responded to different treatments (Figure 6B,D). Grouping of DGGE and PFGE patterns due to glucose and silicate addition was supported statistically (*p* < 0.05, Table 3). Positive correlations (median *p*-value < 0.05) were detected between the bacterial host and the viral communities (0C, 3S, 0CSi), except for the treatment with both carbon and silicate addition (3CSi, median *p*-value 0.08) (Table 4). Correlations were tested with 1000 NMDS permutations of DGGE and PFGE patterns and resulted in a % of *p*-value < 0.05 of 100% (0C), 97.1% (3C), 99.6%, and 27.5% (3CSi) (Table 4). The correlation between the bacterial and viral communities was paralleled by both communities showing significant responses to carbon and silicate treatments (Figure 6B,D, Table 3). However, viral communities responded less to carbon than to silicate, while the opposite was true for the bacterial community (Table 3), with the result that within the mesocosms treated with both C and Si bacterial and viral communities were no longer correlated (Table 4).

## 4. Discussion

Key differences in responses on the level of PFTs in two similar mesocosm experiments (PAME-I and -II) were previously traced to a central role of ciliates, which themselves were top-down controlled by the copepod standing stock [8]. Here, we expand the analysis and demonstrate, first theoretically, how ciliate abundance can be connected to different states of growth limitation of heterotrophic prokaryotes, then experimentally how this is reflected not only in the abundance and activity of heterotrophic prokaryotes but also in the viral communities. 

Our model predicts that high abundance of ciliates (PAME-I) creates a prokaryote community that is organic carbon-limited (OCL) (Figure 1D), which in turn leads to the expectation that glucose addition has a great effect on both abundance and structure of the virus host community. Low ciliate abundances (PAME-II) (Figure 1D), on the other hand, should, according to the model, promote a mineral nutrient-limited (MNL) prokaryote community, giving no expectations of a similar effect when carbon is added. Our experimental results supported our model predictions, with an increase in heterotrophic prokaryote abundance and a statistical supported effect on the bacterial community structure that received additional carbon in PAME-I (Figure 1B and Figure 6B, Table 3). No similar effect or statistical support for changes in the prokaryote abundance or bacterial community structure was seen in PAME-II when carbon was added (Figure 1A and Figure 6A, Table 3). In PAME-I, positive correlations between changes in the bacterial and viral community structure were observed, with one exception (Table 4). A corresponding link was not detected in PAME-II (Table 4). The significant positive correlation between changes in ciliate abundance and virus to prokaryote ratio (VPR) due to organic carbon load (Table 2) further supports our model predictions and demonstrates the strong link between the predator food chain in the marine microbial food web and the activity of viruses. 

Glucose was the key mediator shaping the heterotrophic prokaryote community structure in PAME-I (Table 3, Figure 6B). We also observed, however, an enigmatic effect of silicate on prokaryotes, visible both as an increase in abundance in Si treatments towards the end of the experiment (Figure 1B), and small differences between similar carbon treatments with and without silicate in the community structure of the bacterial community (Figure 6B). Virus abundance increased concomitantly with the prokaryotes (3C+Si) and is indicative of a link between these two populations. Prokaryotes are not expected to be directly influenced by changes in Si concentrations. We believe that the explanation of the enigmatic Si effect on prokaryotes is indirect and linked to the total dominance of the autotrophic community of a single-celled diatom (*Thalassisiosira* sp. 5–10 μm; [10]) small enough to be preyed upon by ciliates in the Si treatments. This led to an increasing ciliate population, which also feed on HNFs and release prokaryotes from predation pressure by HNF, allowing for the observed proliferation of prokaryotes at the end of the experiment [8]. 

As demonstrated in other studies [3,18,19], a statistically supported link was detected between changes in the bacterial host community and changes in the viral community structure (Figure 6B,D, Table 4) in PAME-I. Nevertheless, glucose, which was the main driver for changes in the bacterial community structure, did not seem to influence the viral community to the same extent as silicate (Figure 6D, Table 3). A plausible explanation is that PFGE captures viruses infecting both prokaryotes and eukaryotes [20]. The high abundance of huge viruses (FCM, Figure 4B) and double stranded DNA (dsDNA) viral genomes larger than 250 kb (PFGE, Figure 5) is probably linked to eukaryotic populations, as these generally host bigger viruses than prokaryotes [21,22,23,24,25]. This then explains the low correlation between the bacterial and viral community structure in the 3C+Si tanks (Table 4). *Thalassiosira* sp. diatoms could theoretically host the biggest viruses since they dominated the Si mesocosms [8], but there are presently no reports of huge dsDNA viruses infecting diatoms [26]. We thus deem it more likely that HNFs were hosts to these huge viruses, since the decline in the HNF population [8] coincided with the increase in the huge viral group. This would also be consistent with already characterized giant HNF viruses [24,27]. 

Although the viral community structure in general was most affected by silicate, viral groups I–III were most abundant when carbon was added (Figure 4B). These groups probably comprise the seven PFGE bands with smaller genome size than 100 kb, which were mainly detected at high glucose levels in the period when prokaryote abundance strongly declined (day 5–8) (Figure 5) and may represent viruses able to lyse heterotrophic prokaryotes. 

As important regulators of host diversity and nutrient recycling, viruses are central components of the marine environment and the marine food web. Our results show that the activity of this important regulator in the microbial food web is strongly linked to another top-down mechanism, namely predation and bottom-up factors such as availability of limiting nutrients. To our knowledge, this is the first study that links external trophic interactions in the microbial food web to the internal structure and function of viral and prokaryote communities, using a combination of theoretical modeling and experimental data. Although challenging, understanding these relationships is of particular importance in the rapidly changing Arctic, where growth-limiting factors for prokaryotes have been found to vary [28] and allochthonous import of terrestrial dissolved organic carbon (DOC) from rivers [29], as well as seasonal migration of copepods [30], are central ecosystem characteristics. The present study provides a preliminary understanding of the intricate links between these processes and encourages further cross-scale studies. 

## Figures and Tables

**Table 1:** Symbols and parameter values used in drawing Figure 1D.

Symbol	Meaning	Numerical Value	Unit
**Biomasses**			
*B*	Heterotrophic prokaryotes		nmol-P L^−1^
*H*	Heterotrophic flagellates		nmol-P L^−1^
*C*	Ciliates		nmol-P L^−1^
*A*	Autotrophic flagellates		nmol-P L^−1^
*P*	Free phosphate		nmol-P L^−1^
**Affinities/clearance rates**		**Value at 17 °C**	
*α_B_*	Heterotrophic prokaryote affinity for phosphate	0.08	L nmol-P^−1^h^−1^
*α_A_*	Autotrophic flagellate affinity for phosphate	0.04	L nmol-P^−1^h^−1^
*α_H_*	Heterotrophic flagellate clearance rate for bacteria	0.0015	L nmol-P^−1^h^−1^
*α_C_*	Ciliate clearance rate for flagellates	0.0005	L nmol-P^−1^h^−1^
**Yields**			
*Y_H_*	Heterotrophic flagellate yield on heterotrophic prokaryotes	0.3	nmol-P nmol-P^−1^
*Y_BC_*	Heterotrophic prokaryote yield on DOC		nmol-P nmol-C^−1^
**Conversion factor**			
*α*	P per ciliate	0.00043	nmol-P cell^−1^
**Temperature sensitivity of α-parameters**			
*Q10*		1.3	dimensionless

**Table 2:** Pearson correlation coefficient between ciliate numbers (data from [8]) and viral to prokaryote ratio (VPRs data form Figure 3C,D). 0 × C: no addition of carbon in form of glucose, 3 × C: three Redfield additions of carbon.

	PAME-I		PAME-II	
	0 × C	3 × C	0 × C	3 × C
Persons coefficient (ρ)	−0.175	0.835	0.630	0.783
*p* Value	*p* = 0.678	*p* = 0.00982	*p* = 0.0281	*p* = 0.00258

**Table 3:** Permutational multivariate analysis of variance of band patterns in the denaturing gradient gel electrophoresis (DGGE) and pulsed field gel electrophoresis (PFGE) analysis for bacteria and virus, respectively, from PAME-I and PAME-II mesocosm experiments. Glucose treatments in PAME-I and PAME-II included no addition of carbon in form of glucose (0 × C) and three Redfield additions of carbon (3 × C). Silicate treatment in PAME-I comprised of silicate addition and ambient silicate concentrations. Nitrogen treatment in PAME-II comprised of NH_4_^+^ and NO_3_^−^ addition. All treatments in PAME-II were kept silicate replete. Number of permutations in all analyses: 99999. Residual degrees of freedom: 20 in PAME-I and 12 in PAME-II.

	Treatment	Bacteria Community			Viral Community		
Experiments		F	R^2^	*p*	F	R^2^	*p*
**PAME-I**	Glucose	11.86	0.31	<0.001	7.33	0.11	0.002
	Silicate	5.17	0.14	0.003	38.16	0.56	<0.001
**PAME-II**	Glucose	2.23	0.13	0.07	1.32	0.06	0.3
	Nitrogen	0.63	0.04	0.67	3.53	0.17	0.048

**Table 4:** Median *p*-values, the percentage of significant *p*-values (<0.05) and median correlation values issued from 1000 non-metric multidimensional scaling (NMDS) permutations of denaturing gradient gel electrophoresis (DGGE) and pulsed field gel electrophoresis (PFGE) band patterns are presented for the two different enrichment experiments (PAME-I and -II) and the different tanks. 0 × C: no addition of carbon, 3 × C: three Redfield additions of carbon in form of glucose. Si = Silicate.

	PAME-I				PAME-II			
Statistics	0C	3C	0CSi	3CSi	0CNH_4_	3CNH_4_	0CNO_3_	3CNO_3_
median *p*-value *	0.013	0.01	0.007	0.08	0.083	0.542	0.085	0.125
*p*-values < 0.05 [%] *	100	97.1	99.6	27.5	0	0	0	0
median Procrustes correlation *	0.852	0.878	0.906	0.723	0.963	0.491	0.858	0.945

* Issued from 1000 non-metric multidimensional scaling NMDS permutations.

## Supplementary Materials

The following are available online at www.mdpi.com/1999-4915/9/9/238/s1, Figure S1: title; Minimum model of the photic zone microbial food-web Figure S2: title; Histogram representation of four different viral populations and prokaryotes detected by flow cytometry Supplementary raw data is alsoavailable in the Pangea repository doi:10.1594/PANGAEA.865353.
