# Supplementary material for: The Response of Heterotrophic Prokaryote and Viral Communities to Labile Organic Carbon Inputs Is Controlled by the Predator Food Chain Structure
Source: Viruses. 2017 Aug 23;9(9):238. doi: 10.3390/v9090238 (PMC5618004; doi:10.3390/v9090238)
Supplement: Supplementary file 1 [file viruses-09-00238-s001.pdf]

Suplementary figures:

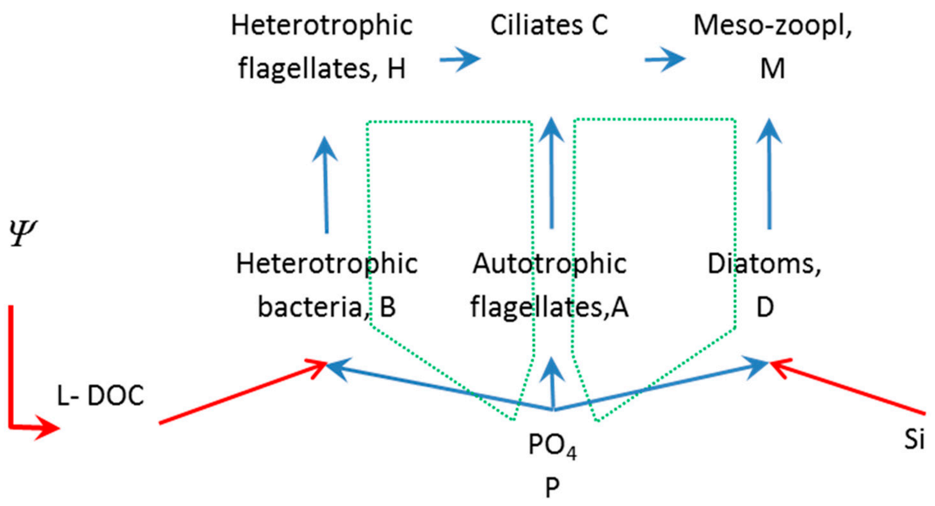

Figure S1. Minimum model of the photic zone microbial food-web constructed as the union of two pentagon-shaped trophic structures (dotted lines). Adapted from Larsen et al. [1].

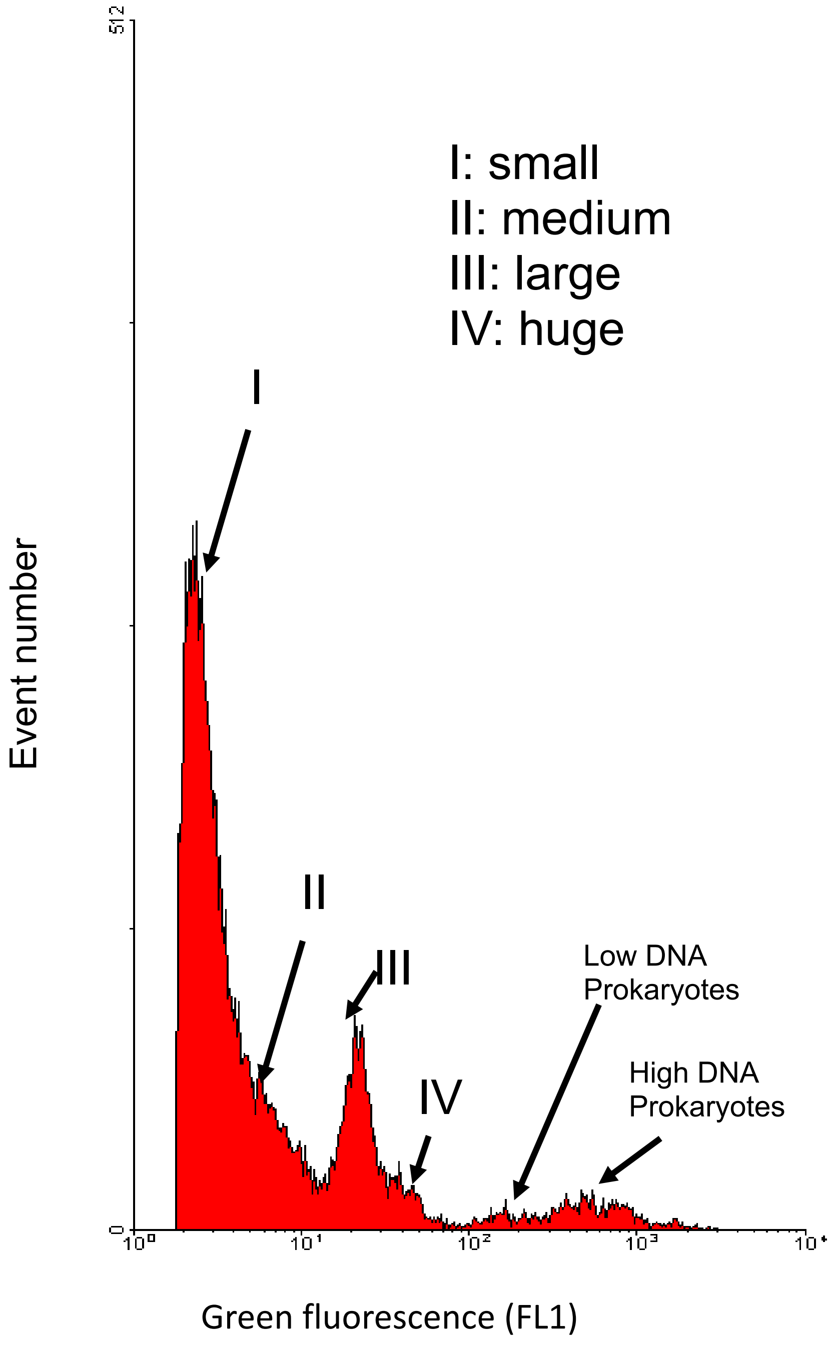

Figure S2. Histogram representation of the four different viral populations and prokaryotes detected by flow cytometry (FMC) showing event number vs green fluorescent signal after staining with SYBR Green I (reflecting the amount of DNA and hence genome sizes). I, Small viruses, II, medium viruses, III, large viruses, IV, huge viruses. The same populations are shown as dot-plots in Figure 3A. The gates used to produce the histograms/dot-plots are set manually but based on the constant appearance of these four groups throughout the study. This is the same procedure used to define autotrophic eukaryotes described for the same experiment in [2] and common to many previous studies using flow cytometry for detection of virus size groups [1, 3, 4].

Supplementary references:

1. Larsen, A.; Egge, J.K.; Nejstgaard, J.C.; Di Capua, I.; Thyrhaug, R.; Bratbak, G.; Thingstad, T.F. Contrasting response to nutrient manipulation in Arctic mesocosms are reproduced by a minimum microbial food web model. *Limnol. Oceanogr.* **2015**, *60,* 360-374.(1)
2. Larsen A.,: Flaten , G.A.F.; Sandaa, R.-A.; Castberg T.; Thyrhaug, R.; Erga S.R.; Jacquet, S.; Bratbak, G. Spring phytoplankton bloom dynamics in Norwegian coastal waters: Microbial community succession and diversity. *Limnol. Oceanogr.***2004.** *49*,180-190.
3. Marie, D.; Brussaard, C.P.D.; Thyrhaug, R.; Bratbak, G.; Vaulot, D. Enumeration of marine viruses in culture and natural samples by flow cytometry. *Appl. Environ. Microbiol.* **1999**, *65*, 45-52.
4. Sandaa R.-A., Larsen A**.** Seasonal variations in viral-host populations in Norwegian coastal waters: Focusing on the cyanophage community infecting marine Synechococcus species. *Appl. Environ. Microbiol.* **2006**.*72,* 4610-4618.
